# Unwanted pregnancy, abortion and suicidal behaviour

**DOI:** 10.1192/j.eurpsy.2025.1326

**Published:** 2025-08-26

**Authors:** N. M. Szeifert, O. Pesthy, X. Gonda

**Affiliations:** 1 Department of Sports Medicine, Semmelweis University; 2 Doctoral School of Psychology, ELTE Eötvös Lóránd University; 3 Institute of Cognitive Neuroscience and Psychology, HUN-REN Research Centre for Natural Sciences; 4 Institute of Psychology, ELTE Eötvös Lóránd University; 5 Department of Psychiatry and Psychotherapy, Semmelweis University, Budapest, Hungary

## Abstract

**Introduction:**

Previous studies have shown that unwanted pregnancy and abortion, particularly in adolescents, are associated with a higher risk of suicidal thoughts and behaviors. Moreover, women with a history of abortion faced a significantly higher risk of various mental health problems, including suicidal behaviors, compared to those who did not undergo abortion. This is often due to a combination of psychological and social pressures. Factors such as the feeling of shame, social isolation, and a sense of being a burden can exacerbate the mental distress that pregnant women or adolescents experience, especially when the pregnancy is unplanned. Additionally, women who attempted suicide often report high levels of stress related to social expectations, family conflict, and lack of support during pregnancy. In some cases, individuals who were born from unwanted pregnancies or whose mother attempted abortions have also shown a higher risk of suicidal behavior later in life. However, our understanding of the risk and protective factors is uncomplete.

**Objectives:**

In our study the connection between unwanted pregnancy, abortion and suicide attempts were analysed and identify their role as potential suicide risk factors.

**Methods:**

*:* Structural clinical interview was used to explore trauma history of induced abortion and unwanted pregnancy. 324 subjects were involved in the analysis. 134 of them with history of suicide, 135 clinical sample without suicide history 55 non-clinical sample. We assessed the moderator effect of the attachment style and childhood trauma using the Adult Attachment Scale (AAS) and the Childhood Trauma Questionnaire (CTQ).

**Results:**

We found no significant effects regarding whether the individual was born from unwanted pregnancies (β = -1.509, p = 0.110, OR = 0.221), and whether they lost their child (β = 0.247, p = 0.892, OR = 0.981). However, whether their mother attempted abortion when pregnant with them have shown a higher risk of suicidal behavior later in life (β = 6.939, p = 0.007, OR = 1031.427). The participants who undergone abortion were also more likely to attempt suicide (β = 2.397, p = 0.011, OR = 10.988). In both cases, childhood trauma was a mediator variable on a significant level (in case of mother’s abortion: β = -0.130, p = 0.005, OR = -2.804) or as a trend (in case of participant’s abortion: β = -0.034, p = 0.050, OR = 0.966).

**Conclusions:**

The perinatal period, particularly for those experiencing unwanted pregnancies, is a critical time for mental health interventions to prevent suicide attempts.

**Disclosure of Interest:**

None Declared

